# Video assisted, transaortic removal of left ventricular thrombus during concurrent cardiac surgery: a case report

**DOI:** 10.1186/s13019-021-01626-4

**Published:** 2021-08-26

**Authors:** Aditya Eranki, Claudia Villanueva, Nicholas Collins, Peng Seah

**Affiliations:** John Hunter Hospital, Newcastle, Australia

**Keywords:** Cardiac surgery, Left ventricular thrombus, Valvular heart surgery, Coronary artery bypass grafting, Video assisted surgery

## Abstract

**Introduction:**

Left ventricular (LV) thrombus is a complication of acute myocardial infarction and is associated with systemic thromboembolism. We describe a trans-aortic endoscopic approach to the removal of an LV thrombus in a patient undergoing concurrent coronary artery bypass grafting and aortic valve replacement.

**Case presentation:**

A 47 year old male presented following an embolic middle cerebral artery stroke and underwent transthoracic echocardiography demonstrating a mobile LV thrombus. Additional investigation revealed a moderately stenosed bicispid aortic valve, two vessel coronary artery disease and ischemic cardiomyopathy. The patient underwent early surgery to reduce the risk of further embolic episodes. A trans-aortic approach was utilized with videoscopy and single shafted instrumentation to aide in removal of the thrombus. The patient then underwent aortic valve replacement and coronary artery bypass grafting.

**Conclusion:**

We report an alternative technique for the removal of a left ventricular thrombus in a patient undergoing concurrent coronary and aortic valve surgery. The transaortic video-assisted approach provided excellent visualisation of the apex and near complete removal of the thrombus without damaging the surrounding trabeculae. The main benefit of this technique is sparing of LV tissue, thereby preserving left ventricular function.

## Introduction

Left ventricular (LV) thrombus is a complication of acute myocardial infarction (MI) and is associated with systemic thromboembolism [1, 2]. It occurs in up to 15% of patients following an ST elevation myocardial infarction (STEMI) and up to 25% of patients following an anterior MI [1]. Systemic thromboembolisation is a feared complication following the development of an LV thrombus and may occur in up to 25% of cases [3]. Stroke may be the first presentation of patients with a LV thrombus. Documented risk factors for the development of LV thrombus following an infarct include large infarct size, severe apical akinesis, LV aneurysm, and anterior MI, which predispose to stasis in the infarcted segment [4].

We describe a patient with severe two vessel disease, ischemic cardiomyopathy and moderate bicuspid aortic stenosis diagnosed after an admission with a left middle cerebral artery (MCA) stroke. Transthoracic echocardiography demonstrated a mobile thrombus. Given the high risk of further embolic sequalae, the patient underwent surgical removal at the time of aortic valve replacement and coronary artery bypass grafting to reduce the patients risk of further stroke.

## Case presentation

### Background

**Fig. 1:**
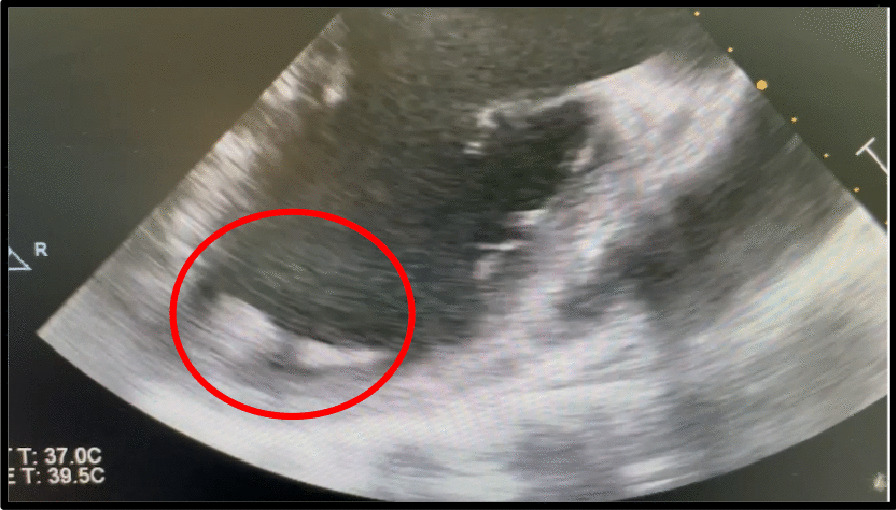
Mid Esophageal Long Axis View demonstrating LV thrombus

A 47-year-old male presented following acute onset right upper limb weakness and expressive dysphasia. The past medical history included well controlled epilepsy and hypertension. There was no history of diabetes mellitus, hypertension or previous smoking. Urgent CT angiography of the head and neck demonstrated a distal left MCA embolic stroke. Transthoracic echocardiography demonstrated left ventricular dilatation with severe systolic dysfunction (left ventricular ejection fraction [LVEF] 25%). Mobile thrombus was noted at the apex of the left ventricle, measuring 14 × 12 mm (Fig. 1). The aortic valve was bicuspid with moderate stenosis and mild valvular regurgitation. The ascending aorta was mildly dilated at less than 40 mm. Subsequent cerebral MR imaging confirmed a left frontal lobe cortical infarct with evidence of focal haemorrhagic transformation and evidence of previous infarction in the MCA territory. The patient was commenced on apixaban and therapy (Bisoprolol and Ramipril) for left ventricular systolic dysfunction. Subsequent angiography demonstrated a significant (80%) proximal LAD stenosis, proximal 90% stenosis of a small non dominant circumflex and 80% stenosis of a large ramus intermediate.

Given the mobile nature of the LV thrombus and the history of stroke, the patient underwent early surgery to reduce the risk of further embolic episodes. The patient proceeded to coronary artery bypass grafting, mechanical aortic valve replacement and resection of the left ventricular thrombus. A repeat CT head scan performed prior to surgery (12 days following the infarction), demonstrated the frontal lobe infarct with no evidence of further haemorrhagic transformation. Apixaban was discontinued and the patient commenced on intravenous heparin in preparation for surgery.

### Surgical procedure

The patient underwent surgery via median sternotomy and the left internal mammary artery (LIMA) was harvested as an in-situ pedicle graft. The left radial was harvested as a free graft. Systemic heparinisation commenced and cardiopulmonary bypass was instituted with bi-caval and distal ascending aortic cannulation. The left ventricle was vented via the right superior pulmonary vein. The aorta was cross clamped and the heart arrested via anterograde and retrograde cold blood cardioplegia. This was repeated at regular intervals to provide myocardial protection.

**Fig. 2:**
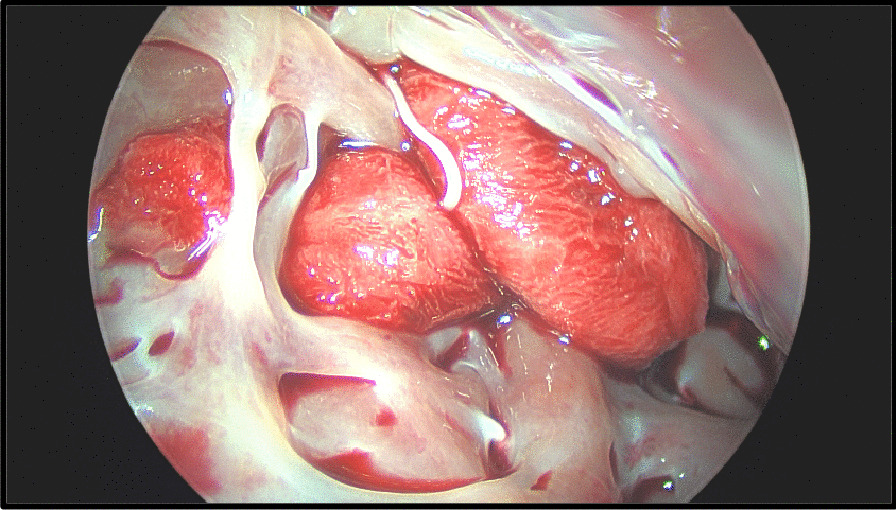
5 mm Videoscopic view of LV thrombus

**Fig. 3:**
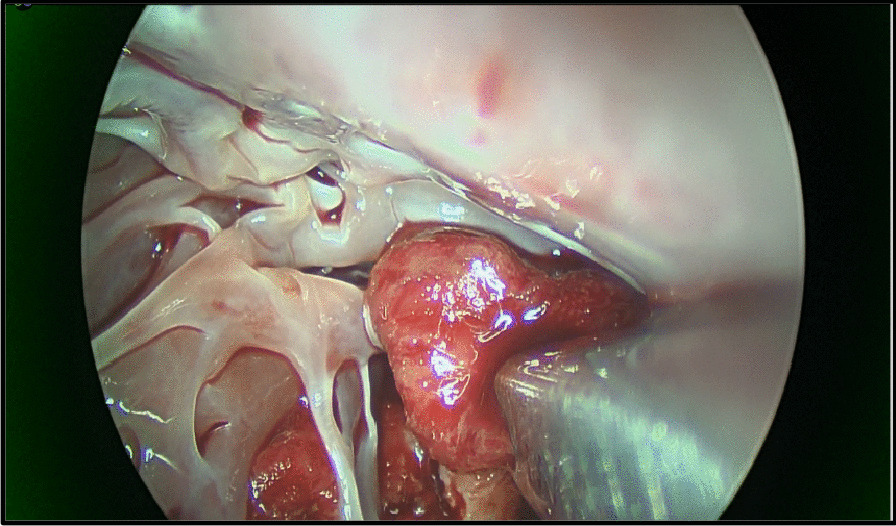
Single shafted instrument removing thrombus

**Fig. 4:**
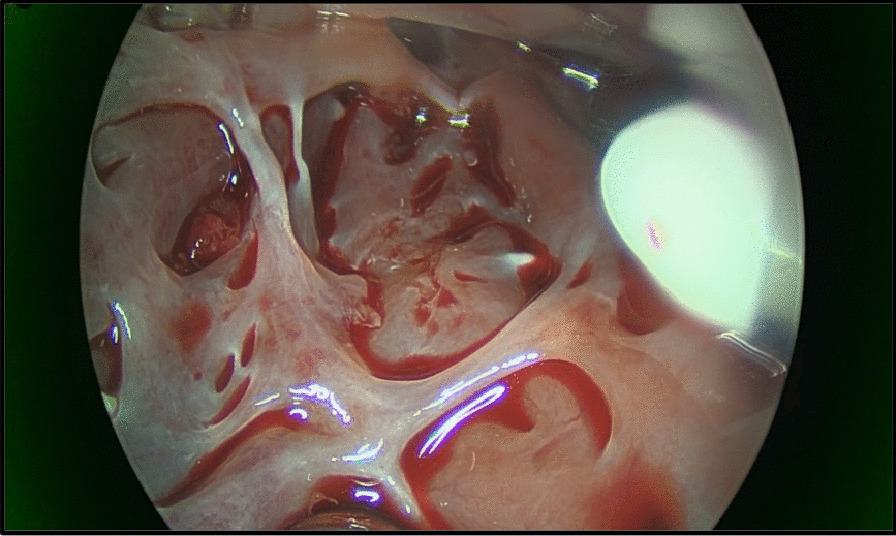
Apical view demonstrating near complete removal

**Fig. 5:**
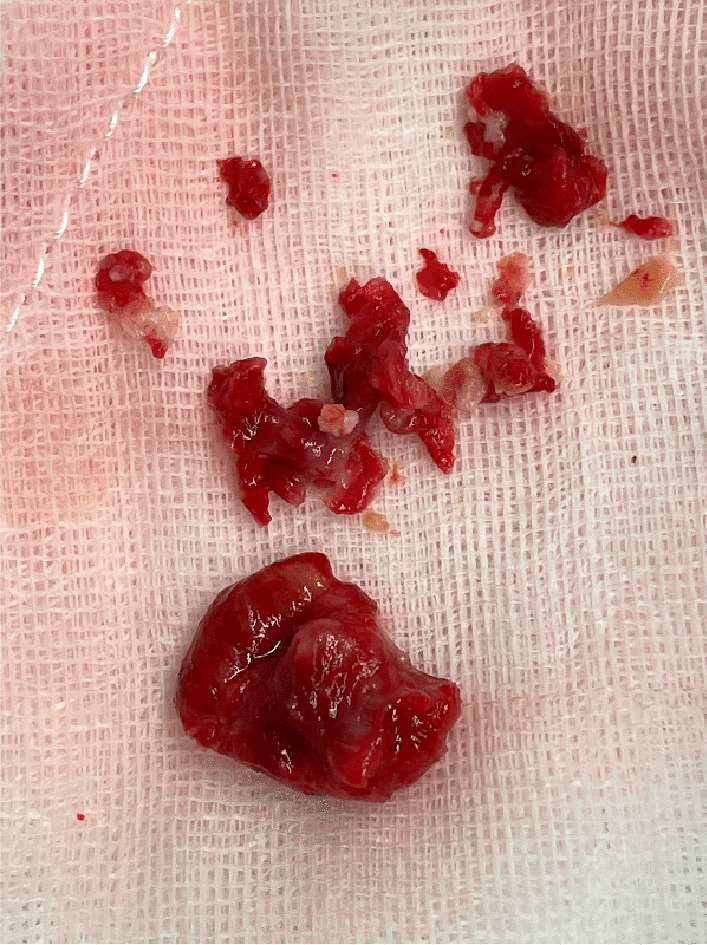
Apical thrombus specimen

Intra operative transesophageal echocardiography confirmed a bicuspid aortic valve with moderate aortic stenosis, severe left ventricular dysfunction and apical left ventricular thrombus with a mobile component (Fig. 1). The aorta was opened in a transverse fashion and the native aortic valve was excised and the annulus decalcified. A 5 mm zero degree Olympus® Videoscope was introduced into the left ventricle with excellent visibility of the left ventricular thrombus assisted by venting of the left ventricle (Fig. 2). With visualisation of the apex under the 5 mm camera, the thrombi were removed using a single shaft grasper and rongeur (Fig. 3). All fresh thrombi were removed, and a fine tipped suction catheter aided in removal of residual thrombus (Figs. 4 and 5). One component of the apical thrombus was firmly embedded within the trabeculae and was not removed as it was felt unlikely to pose an embolic threat (Fig. 4). The left anterior descending artery was bypassed with the LIMA, and the radial artery conduit was used to bypass the ramus branch. The aortic valve was replaced with a 25 mm St Jude mechanical prosthesis and the aortotomy closed. The patient was weaned off cardiopulmonary bypass on first attempt with inotropic support. The patient was discharged day 7 with no complications and maintained on warfarin (target INR of 2 to 3). The LVEF on echocardiography was 45% on discharge.

On review six weeks following surgery there was no evidence of left ventricular thrombus. The aortic valve appeared well seated and there was mild paravalvular aortic regurgitation. There was no residual sequelae from his previous stroke.

## Discussion

Left ventricular thrombus is a recognised complication following acute MI, and can occur as frequently as in 25% of patients following an anterior MI [1]. Transthoracic echocardiography is typically the screening modality of choice and should be done within 24 h of admission for those at high risk of LV thrombus [1]. If the apex is poorly visualised, anterior or apical regional wall abnormalities are present then contrast TTE or cardiac MRI should be considered [1]. Stroke is a recognized complication following LV thrombus and a retrospective study by Leow et al. demonstrated an incidence of 12% [5]. Treatment of LV thrombus revolves around anticoagulation and close follow up with guidelines advocating for 3 months following detection. When a patient is undergoing concurrent cardiac surgery or where the thrombus is at high risk of embolization, surgical removal may be warranted [3, 6]. Warfarin is the mainstay of anticoagulation [7]. Evidence suggests that novel oral anticoagulation is associated with higher rates of stroke and systemic embolization [7].

Removal of these thrombi via a left ventriculotomy is feasible, however cases of this approach have been reserved for patients with a concurrent ventricular aneurysm requiring repair [8]. The concern with a left ventriculotomy is further depression of left ventricular function, friability of tissue following an acute myocardial infarction and arrhythmogenic potential post operatively. These concerns were relevant to our patient given his pre-existing left ventricular dysfunction. An alternate approach via the left atrium has been described by Tanaka et al. and allows extraction of a larger thrombus than the trans-aortic approach [9]. Pitfalls include limited room for manipulation of the thrombus and should be reserved for those which are felt to be loosely connected with a narrow stalk [10].

Case studies report the use of videoscopy to visualise intra-cardiac structures and to assist in excision of left ventricular lesions as a viable alternative to a ventriculotomy. A systematic review by Soylu et al. summarises a total of 34 studies incorporating 54 patients where left ventricular cardiac tumours were removed with the use of videoscopic devices [11]. The majority of these occurred in conjunction with other cardiac surgical procedures, with complete resection achieved in all cases [11]. Takusube et al. first described the transaortic video assisted removal of a left ventricular thrombus via the use of a videoscope, reporting excellent visualisation of thrombus and satisfactory postoperative outcome [12]. Other cases using this approach for a left ventricular thrombus also report excellent visualisation of the apex and excellent postoperative outcomes [13–16]. Of note Williamson et al. describe a case of a LV thrombus resection in a patient undergoing concurrent cardiac and aortic valve surgery [13]. They used a 5 mm camera through the aortic annulus and described ease of access to the apex of the LV via curved Randall stone forceps [13]. In our case, the 5 mm scope could be placed across the annulus allowing for the passage of single shafted instrumentation. The scope was also useful as a retractor to obtain better access to the thrombus at the LV apex. In cases where the aortic valve is not replaced, it may be feasible to pass the 5 mm scope across the valve without traumatising the valve with the advantage that it allows for room for the passage of further instrumentation.

## Conclusion

We report an alternative technique for the removal of a left ventricular thrombus in a patient undergoing concurrent coronary and aortic valve surgery. The transaortic video-assisted approach provided excellent visualisation of the apex and near complete removal of the thrombus without damaging the surrounding trabeculae. The main benefit of this technique is sparing of LV tissue, thereby preserving left ventricular function.

## Data Availability

Data sharing is not applicable to this article as no datasets were generated or analysed during the current study.
